# ID 185 - Inovação na Residência Multiprofissional: Eixo Transversal do Núcleo de Avaliação de Tecnologias em Saúde do HU-UFJF

**DOI:** 10.5327/2237-9622.2025.v34s1.185

**Published:** 2025-11-25

**Authors:** Erika Maria Henriques Monteiro, Luciana de Sousa Santos Costa, Aline Mota Freitas Matos, Maurílio de Souza Cazarim, Igor Rosa Meurer

**Keywords:** Avaliação de Tecnologias em Saúde, residência multiprofissional, Nats, evidências científicas, gestão hospitalar

## Abstract

**Introdução:**

A necessidade de decisões baseadas em evidências no ambiente hospitalar tem crescido, especialmente no contexto de incorporação de novas tecnologias. No Hospital Universitário da Universidade Federal de Juiz de Fora (HU-UFJF), o Núcleo de Avaliação de Tecnologias em Saúde (Nats) foi integrado ao Programa de Residência Multiprofissional como um eixo transversal, criado para capacitar residentes em Avaliação de Tecnologias em Saúde (ATS). Esse eixo visa promover o uso racional de tecnologias e contribuir para a tomada de decisões clínicas e administrativas embasadas em evidências. O objetivo geral é fortalecer a formação dos residentes e aprimorar a eficiência da gestão hospitalar, com metas específicas voltadas ao desenvolvimento de habilidades em busca de evidências científicas, uso de ferramentas tecnológicas e avaliação crítica de tecnologias.

**Método:**

A implementação do Eixo Nats seguiu quatro etapas principais:

- capacitação teórica e prática em ATS para residentes e preceptores, com os seguintes módulos:
  – apresentação do Nats, princípios de ATS e saúde baseada em evidências (6h);
  – ferramentas como Rayyan, Mendeley e normas acadêmicas (9h);
  – fundamentos de estatística aplicada e avaliação crítica de artigos (9h);
  – introdução à farmacoeconomia e sua aplicação na ATS (6h);
  – avaliação da qualidade de evidências com GRADE e ROB 2.0 (6h);
  – fluxo de submissão de projetos de pesquisa no HU-UFJF e Plataforma Brasil (6h);
  – recapitulação e avaliação final (3h).
- aplicação prática com a participação dos residentes na elaboração de pareceres técnicos e relatórios de miniATS;
- monitoramento dos impactos das tecnologias avaliadas na eficiência hospitalar e na segurança dos pacientes;
- avaliação contínua dos residentes por meio de testes teóricos e análise de desempenho nas atividades práticas.

**Resultados:**

A prática resultou em um aumento significativo na capacidade de análise crítica dos residentes. O eixo proporcionou um ambiente colaborativo, envolvendo profissionais de diversas áreas da saúde, como farmácia, enfermagem, nutrição, fisioterapia e psicologia. Cada ciclo é composto por 17 encontros semanais de três horas, totalizando 51 horas de formação teórica e prática ao longo de quatro meses.

**Conclusão:**

O Eixo Nats impactou positivamente tanto a formação dos residentes quanto a eficiência hospitalar. A prática demonstrou uma gestão mais eficiente e segura das tecnologias no HU-UFJF, sendo inovadora por promover interdisciplinaridade e capacitação tecnológica. O sucesso do modelo sugere sua replicabilidade em outros hospitais de ensino, gerando benefícios similares em termos de eficiência e de qualificação profissional. Conclui-se que a prática é essencial para a formação contínua dos profissionais de saúde e para o fortalecimento da gestão baseada em evidências.

